# Bis[*O*-methyl (4-eth­oxy­phen­yl)dithio­phospho­nato-κ^2^ *S*,*S*′]nickel(II)

**DOI:** 10.1107/S1600536812046314

**Published:** 2012-11-14

**Authors:** Shirveen Sewpersad, Bernard Omondi, Werner E. Van Zyl

**Affiliations:** a School of Chemistry and Physics, University of KwaZulu-Natal, Westville Campus, Private Bag X54001, Durban 4000, South Africa

## Abstract

In the title compound, [Ni(C_9_H_12_O_2_PS_2_)_2_], the Ni^II^ atom resides on an inversion center and is coordinated by four S atoms [Ni—S = 2.2328 (4) and 2.2455 (3) Å] in a distorted square-planar geometry [S—Ni—S = 88.443 (13) and 91.557 (13)°]. In the crystal, mol­ecules related by translation in [110] are linked into chains *via* weak C—H⋯O inter­actions. The crystal packing exhibits short inter­molecular S⋯S contacts of 3.3366 (5) Å.

## Related literature

For information on dithio­phospho­nate compounds, see: Van Zyl & Fackler (2000 ▶); Van Zyl (2010 ▶); Van Zyl & Woollins (2012 ▶). For related structures of nickel(II) dithio­phospho­nate complexes, see: Hartung (1967 ▶); Liu *et al.* (2004 ▶); Gray *et al.* (2004 ▶); Aragoni *et al.* (2007 ▶); Arca *et al.* (1997 ▶); Özcan *et al.* (2002 ▶).
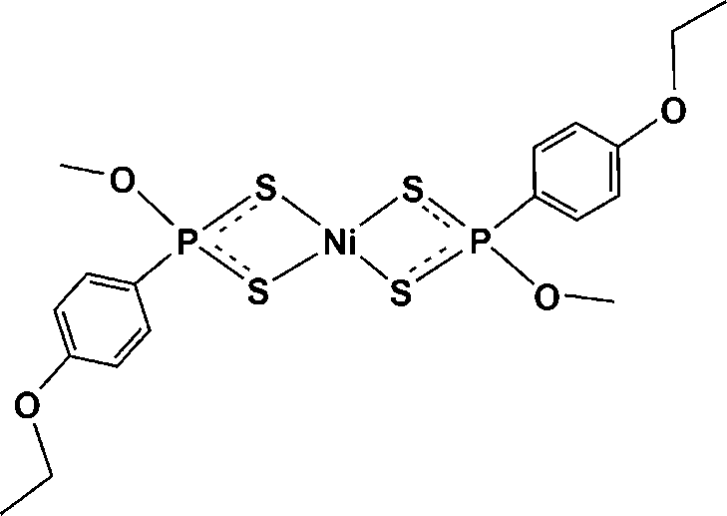


## Experimental

### 

#### Crystal data

[Ni(C_9_H_12_O_2_PS_2_)_2_]
*M* *_r_* = 553.26
Monoclinic, 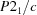

*a* = 13.5866 (5) Å
*b* = 6.4212 (2) Å
*c* = 14.1047 (5) Å
β = 109.389 (2)°
*V* = 1160.74 (7) Å^3^
*Z* = 2
Mo *K*α radiation
μ = 1.36 mm^−1^
*T* = 173 K
0.43 × 0.31 × 0.24 mm

#### Data collection

Bruker SMART APEXII CCD diffractometer
Absorption correction: multi-scan (*SADABS*; Bruker, 2008 ▶) *T*_min_ = 0.593, *T*_max_ = 0.737
19824 measured reflections
2850 independent reflections
2609 reflections with *I* > 2σ(*I*)
*R*_int_ = 0.036

#### Refinement

*R*[*F* ^2^ > 2σ(*F* ^2^)] = 0.023
*wR*(*F* ^2^) = 0.062
*S* = 1.07
2850 reflections
135 parameters
2 restraints
H-atom parameters constrained
Δρ_max_ = 0.56 e Å^−3^
Δρ_min_ = −0.44 e Å^−3^

### 

Data collection: *APEX2* (Bruker, 2008 ▶); cell refinement: *SAINT-Plus* (Bruker, 2008 ▶); data reduction: *SAINT-Plus* and *XPREP* (Bruker, 2008 ▶); program(s) used to solve structure: *SHELXS97* (Sheldrick, 2008 ▶); program(s) used to refine structure: *SHELXL97* (Sheldrick, 2008 ▶); molecular graphics: *SHELXTL* (Sheldrick, 2008 ▶); software used to prepare material for publication: *SHELXL97*.

## Supplementary Material

Crystal structure: contains datablock(s) I, global. DOI: 10.1107/S1600536812046314/cv5358sup1.cif

Structure factors: contains datablock(s) I. DOI: 10.1107/S1600536812046314/cv5358Isup2.hkl

Additional supplementary materials:  crystallographic information; 3D view; checkCIF report

## Figures and Tables

**Table 1:** Hydrogen-bond geometry (Å, °)

*D*—H⋯*A*	*D*—H	H⋯*A*	*D*⋯*A*	*D*—H⋯*A*
C3—H3⋯O1^i^	0.95	2.57	3.5123 (18)	171

Symmetry code: (i) 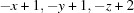.

**Fig. 1.**
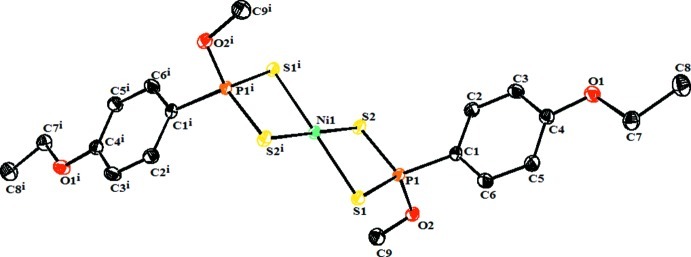
The molecular structure of the title complex showing the atomic numbering and 50% probability displacement ellispoids [symmetry code: (i)-x + 2,-y + 2,-z + 2]. H atoms omitted for clarity.

## supplementary crystallographic information

### Comment

The phosphor-1,1-dithiolate class of compounds is the heavier and softer congener of the popular phosphonate derivatives. It contains the S_2_P functionality as a common feature and several sub-categories are known which include the dithiophosphato [S_2_P(OR)_2_]¯, (*R* = typically alkyl), dithiophosphinato [S_2_P*R*_2_]¯ (*R* = alkyl or aryl), and dithiophosphonato [S_2_PR(OR')]¯, (*R* = typically aryl or ferrocenyl, *R*' = alkyl) monoanionic ligands. The latter may be described as a hybrid of the former two, and are also much less developed. Amongst all metals involved in the coordination chemistry of dithiophosphonato ligands, however, nickel(II) is by far the best represented (Van Zyl & Woollins, 2012) with the first X-ray structural report of a nickel(II) dithiophosphonate complex reported more than four decades ago (Hartung, 1967). The title complex, (I), was formed from the reaction between NiCl_2_.6H_2_0 and the ammonium salt of [S_2_P(OMe)(4-C_6_H_4_OEt)] (molar ratio 1:2) in an aqueous/methanolic solution, the NH_4_Cl by-product was dissolved and the precipitated product filtered off and washed with water. General and convenient methods to prepare dithiophosphonate salt derivatives have been reported (Van Zyl & Fackler, 2000).

The structure of (I) (Fig. 1) does not differ significantly from related Ni(II) complexes previously reported (Aragoni *et al.*, 2007; Arca *et al.* (1997); Gray *et al.* (2004); Liu *et al.* (2004); Özcan *et al.*, 2002). The Ni atom in (I) resides on an inversion center and is coordinated by four S atoms [Ni—S 2.2328 (4), 2.2455 (3) Å] in a distorted square-planar geometry [S—Ni—S 88.443 (13), 91.557 (13)°]. Molecules related by translation in [110] are linked into chains *via* weak C—H···O interactions (Table 1). The crystal packing exhibits short intermolecular S···S contacts of 3.3366 (5) Å.

### Experimental

A colorless methanol (40 ml) solution of NH_4_[S_2_P(OMe)(4-C_6_H_4_OEt)] (1.044 g, 4.474 mmol) was prepared. A second green solution of NiCl_2_.6H_2_0 (540 mg, 2.272 mmol) in deionized water (20 ml) was prepared, and added to the colorless solution with stirring over a period of 5 min. This resulted in a purple precipitate indicating the formation of the title complex. The precipitate was collected by vacuum filtration, washed with water (3 *x* 10 ml) and allowed to dry under vacuum for a period of 3 hrs, yielding a dry, free-flowing purple powder. Purple crystals suitable for X-ray analysis were grown by the slow diffusion of hexane into a dichloromethane solution of the title complex. Yield: 1.004 g, 41%. *M*.p. 168°C.

^31^P NMR (CDCl_3_): δ (p.p.m.): 104.56. ^1^H NMR (CDCl_3_): δ (p.p.m.): 7.94 (2*H*, dd, J(^31^P-^1^H) = 12.76 Hz, J(^1^H -^1^H) = 10.08 Hz, *o*-ArH), 6.95 (2*H*, dd, J(^31^P-^1^H) = 8.76 Hz, J(^1^H-^1^H) = 3.08 Hz, *m*-ArH), 4.07 (2*H*, quart, J(^1^H-^1^H) = 6.96 Hz, ArOCH_2_), 3.96 (3*H*, d, J(^31^P-^1^H) = 14.8 Hz, POCH_3_), 1.41 (3*H*, t, J(^1^H-^1^H) = 6.98 Hz, ArOCH_2_CH_3_). ^13^C NMR (CDCl_3_): δ (p.p.m.): 162.64 (*p*-ArC), 131.78 (*m*-ArC), 128.68 (Ar—C*_ipso_*), 114.62 (*o*-ArC), 63.99 (ArOCH_2_), 52.73 (OCH_3_), 14.85 (ArOCH_2_CH_3_).

### Refinement

All hydrogen atoms were found in the difference electron density maps, then placed in idealized positions (C—H = 0.95–1.00 Å) and refined as riding, with U_iso_(H) = 1.2-1.5 U_eq_(C).

### Crystal data

**Table:**

[Ni(C_9_H_12_O_2_PS_2_)_2_]	*F*(000) = 572
*M**_r_* = 553.26	*D*_x_ = 1.583 Mg m^−^^3^
Monoclinic, *P*2_1_/*c*	Mo *K*α radiation, λ = 0.71073 Å
Hall symbol: -P 2ybc	Cell parameters from 19824 reflections
*a* = 13.5866 (5) Å	θ = 2.9–28.4°
*b* = 6.4212 (2) Å	µ = 1.36 mm^−^^1^
*c* = 14.1047 (5) Å	*T* = 173 K
β = 109.389 (2)°	Block, purple
*V* = 1160.74 (7) Å^3^	0.43 × 0.31 × 0.24 mm
*Z* = 2	

### Data collection

**Table:**

Bruker SMART APEXII CCD diffractometer	2850 independent reflections
Radiation source: fine-focus sealed tube	2609 reflections with *I* > 2σ(*I*)
Graphite monochromator	*R*_int_ = 0.036
φ and ω scans	θ_max_ = 28.4°, θ_min_ = 2.9°
Absorption correction: multi-scan (*SADABS*; Bruker, 2008)	*h* = −18→17
*T*_min_ = 0.593, *T*_max_ = 0.737	*k* = −8→8
19824 measured reflections	*l* = −18→18

### Refinement

**Table:**

Refinement on *F*^2^	Primary atom site location: structure-invariant direct methods
Least-squares matrix: full	Secondary atom site location: difference Fourier map
*R*[*F*^2^ > 2σ(*F*^2^)] = 0.023	Hydrogen site location: inferred from neighbouring sites
*wR*(*F*^2^) = 0.062	H-atom parameters constrained
*S* = 1.07	*w* = 1/[σ^2^(*F*_o_^2^) + (0.0281*P*)^2^ + 0.7956*P*] where *P* = (*F*_o_^2^ + 2*F*_c_^2^)/3
2850 reflections	(Δ/σ)_max_ = 0.001
135 parameters	Δρ_max_ = 0.56 e Å^−^^3^
2 restraints	Δρ_min_ = −0.44 e Å^−^^3^

### Special details

**Table:**

Geometry. All e.s.d.'s (except the e.s.d. in the dihedral angle between two l.s. planes) are estimated using the full covariance matrix. The cell e.s.d.'s are taken into account individually in the estimation of e.s.d.'s in distances, angles and torsion angles; correlations between e.s.d.'s in cell parameters are only used when they are defined by crystal symmetry. An approximate (isotropic) treatment of cell e.s.d.'s is used for estimating e.s.d.'s involving l.s. planes.
Refinement. Refinement of *F*^2^ against ALL reflections. The weighted *R*-factor *wR* and goodness of fit *S* are based on *F*^2^, conventional *R*-factors *R* are based on *F*, with *F* set to zero for negative *F*^2^. The threshold expression of *F*^2^ > σ(*F*^2^) is used only for calculating *R*-factors(gt) *etc*. and is not relevant to the choice of reflections for refinement. *R*-factors based on *F*^2^ are statistically about twice as large as those based on *F*, and *R*- factors based on ALL data will be even larger.

### Fractional atomic coordinates and isotropic or equivalent isotropic displacement parameters (Å^2^)

**Table:**

	*x*	*y*	*z*	*U*_iso_*/*U*_eq_	
C1	0.70393 (11)	0.8288 (2)	0.85131 (10)	0.0149 (3)	
C2	0.68704 (11)	0.6590 (2)	0.90614 (11)	0.0175 (3)	
H2	0.7398	0.5564	0.9306	0.021*	
C3	0.59371 (12)	0.6398 (2)	0.92488 (12)	0.0196 (3)	
H3	0.5823	0.5237	0.9617	0.024*	
C4	0.51619 (11)	0.7913 (2)	0.88969 (11)	0.0167 (3)	
C5	0.53108 (12)	0.9588 (3)	0.83320 (12)	0.0202 (3)	
H5	0.4777	1.0598	0.8075	0.024*	
C6	0.62531 (12)	0.9759 (3)	0.81507 (12)	0.0211 (3)	
H6	0.6362	1.0905	0.7771	0.025*	
C7	0.34352 (11)	0.9068 (2)	0.87693 (12)	0.0199 (3)	
H7A	0.3191	0.9120	0.8026	0.024*	
H7B	0.3662	1.0480	0.9033	0.024*	
C8	0.25769 (12)	0.8303 (3)	0.91356 (13)	0.0258 (3)	
H8A	0.2401	0.6862	0.8915	0.039*	
H8B	0.1959	0.9186	0.8859	0.039*	
H8C	0.2812	0.8364	0.9871	0.039*	
C9	0.88481 (12)	0.8281 (3)	0.67579 (12)	0.0243 (3)	
H9A	0.9184	0.9650	0.6891	0.036*	
H9B	0.8562	0.8052	0.6031	0.036*	
H9C	0.9363	0.7197	0.7065	0.036*	
O1	0.42818 (8)	0.76065 (18)	0.91366 (8)	0.0210 (2)	
O2	0.80067 (8)	0.81965 (17)	0.71841 (7)	0.0172 (2)	
P1	0.82716 (3)	0.87053 (6)	0.83456 (3)	0.01290 (9)	
S1	0.88018 (3)	1.16115 (5)	0.87163 (3)	0.01505 (9)	
S2	0.94269 (3)	0.69659 (5)	0.92450 (3)	0.01547 (9)	
Ni1	1.0000	1.0000	1.0000	0.01236 (8)	

### Atomic displacement parameters (Å^2^)

**Table:**

	*U*^11^	*U*^22^	*U*^33^	*U*^12^	*U*^13^	*U*^23^
C1	0.0126 (6)	0.0163 (7)	0.0142 (6)	−0.0026 (5)	0.0024 (5)	−0.0008 (5)
C2	0.0168 (7)	0.0162 (7)	0.0186 (7)	0.0021 (5)	0.0050 (5)	0.0025 (6)
C3	0.0200 (7)	0.0181 (7)	0.0218 (7)	0.0007 (6)	0.0085 (6)	0.0061 (6)
C4	0.0156 (6)	0.0187 (7)	0.0157 (6)	−0.0013 (6)	0.0050 (5)	−0.0007 (6)
C5	0.0152 (7)	0.0194 (7)	0.0246 (7)	0.0024 (6)	0.0050 (6)	0.0065 (6)
C6	0.0177 (7)	0.0201 (7)	0.0252 (7)	0.0005 (6)	0.0067 (6)	0.0088 (6)
C7	0.0171 (7)	0.0192 (7)	0.0239 (7)	0.0027 (6)	0.0074 (6)	−0.0006 (6)
C8	0.0209 (7)	0.0271 (9)	0.0324 (9)	0.0015 (6)	0.0127 (7)	−0.0004 (7)
C9	0.0219 (7)	0.0328 (9)	0.0212 (7)	0.0004 (7)	0.0113 (6)	−0.0025 (7)
O1	0.0176 (5)	0.0231 (6)	0.0251 (5)	0.0041 (4)	0.0107 (4)	0.0069 (5)
O2	0.0164 (5)	0.0212 (5)	0.0133 (5)	−0.0019 (4)	0.0038 (4)	−0.0016 (4)
P1	0.01195 (16)	0.01266 (17)	0.01283 (16)	−0.00089 (13)	0.00241 (13)	0.00025 (13)
S1	0.01442 (16)	0.01179 (16)	0.01655 (16)	−0.00085 (12)	0.00194 (13)	0.00125 (13)
S2	0.01453 (16)	0.01163 (17)	0.01733 (16)	0.00058 (12)	0.00138 (13)	−0.00038 (13)
Ni1	0.01162 (12)	0.01061 (13)	0.01350 (12)	−0.00046 (9)	0.00235 (9)	0.00017 (9)

### Geometric parameters (Å, º)

**Table:**

C1—C6	1.390 (2)	C8—H8A	0.9800
C1—C2	1.399 (2)	C8—H8B	0.9800
C1—P1	1.7871 (14)	C8—H8C	0.9800
C2—C3	1.384 (2)	C9—O2	1.4585 (17)
C2—H2	0.9500	C9—H9A	0.9800
C3—C4	1.398 (2)	C9—H9B	0.9800
C3—H3	0.9500	C9—H9C	0.9800
C4—O1	1.3606 (17)	O2—P1	1.5902 (10)
C4—C5	1.393 (2)	P1—S2	1.9996 (5)
C5—C6	1.391 (2)	P1—S1	2.0061 (5)
C5—H5	0.9500	P1—Ni1	2.8306 (4)
C6—H6	0.9500	S1—Ni1	2.2455 (3)
C7—O1	1.4408 (18)	S2—Ni1	2.2328 (4)
C7—C8	1.506 (2)	Ni1—S2^i^	2.2328 (4)
C7—H7A	0.9900	Ni1—S1^i^	2.2455 (3)
C7—H7B	0.9900	Ni1—P1^i^	2.8306 (4)
			
C6—C1—C2	119.07 (13)	O2—C9—H9C	109.5
C6—C1—P1	119.25 (11)	H9A—C9—H9C	109.5
C2—C1—P1	121.52 (11)	H9B—C9—H9C	109.5
C3—C2—C1	120.23 (14)	C4—O1—C7	118.63 (12)
C3—C2—H2	119.9	C9—O2—P1	118.47 (9)
C1—C2—H2	119.9	O2—P1—C1	101.76 (6)
C2—C3—C4	120.03 (14)	O2—P1—S2	113.60 (4)
C2—C3—H3	120.0	C1—P1—S2	113.88 (5)
C4—C3—H3	120.0	O2—P1—S1	113.49 (4)
O1—C4—C5	124.08 (14)	C1—P1—S1	112.10 (5)
O1—C4—C3	115.59 (13)	S2—P1—S1	102.47 (2)
C5—C4—C3	120.33 (13)	O2—P1—Ni1	138.74 (4)
C6—C5—C4	118.91 (14)	C1—P1—Ni1	119.49 (5)
C6—C5—H5	120.5	S2—P1—Ni1	51.639 (13)
C4—C5—H5	120.5	S1—P1—Ni1	51.985 (12)
C1—C6—C5	121.39 (14)	P1—S1—Ni1	83.279 (16)
C1—C6—H6	119.3	P1—S2—Ni1	83.755 (17)
C5—C6—H6	119.3	S2^i^—Ni1—S2	180.0
O1—C7—C8	106.33 (13)	S2^i^—Ni1—S1	91.557 (13)
O1—C7—H7A	110.5	S2—Ni1—S1	88.443 (13)
C8—C7—H7A	110.5	S2^i^—Ni1—S1^i^	88.443 (13)
O1—C7—H7B	110.5	S2—Ni1—S1^i^	91.557 (13)
C8—C7—H7B	110.5	S1—Ni1—S1^i^	180.0
H7A—C7—H7B	108.7	S2^i^—Ni1—P1^i^	44.606 (11)
C7—C8—H8A	109.5	S2—Ni1—P1^i^	135.394 (11)
C7—C8—H8B	109.5	S1—Ni1—P1^i^	135.263 (12)
H8A—C8—H8B	109.5	S1^i^—Ni1—P1^i^	44.737 (12)
C7—C8—H8C	109.5	S2^i^—Ni1—P1	135.394 (11)
H8A—C8—H8C	109.5	S2—Ni1—P1	44.606 (11)
H8B—C8—H8C	109.5	S1—Ni1—P1	44.737 (12)
O2—C9—H9A	109.5	S1^i^—Ni1—P1	135.263 (12)
O2—C9—H9B	109.5	P1^i^—Ni1—P1	180.0
H9A—C9—H9B	109.5		
			
C6—C1—C2—C3	−0.8 (2)	C1—P1—S2—Ni1	−109.65 (5)
P1—C1—C2—C3	174.69 (12)	S1—P1—S2—Ni1	11.639 (18)
C1—C2—C3—C4	−0.5 (2)	P1—S2—Ni1—S2^i^	67 (100)
C2—C3—C4—O1	−178.75 (14)	P1—S2—Ni1—S1	−10.140 (16)
C2—C3—C4—C5	1.9 (2)	P1—S2—Ni1—S1^i^	169.860 (16)
O1—C4—C5—C6	178.78 (14)	P1—S2—Ni1—P1^i^	180.0
C3—C4—C5—C6	−1.9 (2)	P1—S1—Ni1—S2^i^	−169.884 (16)
C2—C1—C6—C5	0.7 (2)	P1—S1—Ni1—S2	10.116 (16)
P1—C1—C6—C5	−174.82 (13)	P1—S1—Ni1—S1^i^	−11 (100)
C4—C5—C6—C1	0.6 (2)	P1—S1—Ni1—P1^i^	180.0
C5—C4—O1—C7	1.5 (2)	O2—P1—Ni1—S2^i^	97.35 (7)
C3—C4—O1—C7	−177.89 (13)	C1—P1—Ni1—S2^i^	−81.64 (6)
C8—C7—O1—C4	178.93 (13)	S2—P1—Ni1—S2^i^	180.000 (1)
C9—O2—P1—C1	−177.47 (12)	S1—P1—Ni1—S2^i^	14.48 (2)
C9—O2—P1—S2	−54.64 (12)	O2—P1—Ni1—S2	−82.65 (7)
C9—O2—P1—S1	61.90 (12)	C1—P1—Ni1—S2	98.36 (6)
C9—O2—P1—Ni1	3.43 (15)	S1—P1—Ni1—S2	−165.52 (2)
C6—C1—P1—O2	−74.92 (13)	O2—P1—Ni1—S1	82.87 (7)
C2—C1—P1—O2	109.62 (12)	C1—P1—Ni1—S1	−96.12 (6)
C6—C1—P1—S2	162.44 (11)	S2—P1—Ni1—S1	165.52 (2)
C2—C1—P1—S2	−13.02 (14)	O2—P1—Ni1—S1^i^	−97.13 (7)
C6—C1—P1—S1	46.67 (13)	C1—P1—Ni1—S1^i^	83.88 (6)
C2—C1—P1—S1	−128.78 (11)	S2—P1—Ni1—S1^i^	−14.48 (2)
C6—C1—P1—Ni1	104.40 (12)	S1—P1—Ni1—S1^i^	180.0
C2—C1—P1—Ni1	−71.06 (13)	O2—P1—Ni1—P1^i^	−20 (100)
O2—P1—S1—Ni1	−134.48 (4)	C1—P1—Ni1—P1^i^	161 (100)
C1—P1—S1—Ni1	110.92 (5)	S2—P1—Ni1—P1^i^	63 (100)
S2—P1—S1—Ni1	−11.583 (18)	S1—P1—Ni1—P1^i^	−102 (100)
O2—P1—S2—Ni1	134.46 (5)		

Symmetry code: (i) −*x*+2, −*y*+2, −*z*+2.

### Hydrogen-bond geometry (Å, º)

**Table:**

*D*—H···*A*	*D*—H	H···*A*	*D*···*A*	*D*—H···*A*
C3—H3···O1^ii^	0.95	2.57	3.5123 (18)	171

Symmetry code: (ii) −*x*+1, −*y*+1, −*z*+2.
